# Novel pathways of HIV latency reactivation revealed by integrated analysis of transcriptome and target profile of bryostatin

**DOI:** 10.1038/s41598-020-60614-1

**Published:** 2020-02-26

**Authors:** Bing-xiang Li, Han Zhang, Yubin Liu, Ya Li, Jun-juan Zheng, Wen-Xing Li, Kai Feng, Ming Sun, Shao-Xing Dai

**Affiliations:** 1 Institute of Medical Biology, Peking Union Medical College and Chinese Academy of Medical Sciences, Kunming, China; 2 Yunnan Key Laboratory of Primate Biomedicine Research, Institute of Primate Translational Medicine, Kunming University of Science and Technology, Kunming, 650500 Yunnan China; 3 Department of Infectious Diseases, Peking Union Medical College Hospital, Peking Union Medical College and Chinese Academy of Medical Sciences, Beijing, China; 4 Yunnan Key Laboratory of Laboratory Medicine, Yunnan Institute of Laboratory Diagnosis, Department of Clinical Laboratory, The First Affiliated Hospital of Kunming Medical University, Kunming, 650032 Yunnan China; 5 State Key Laboratory of Genetic Resources and Evolution, Kunming Institute of Zoology, Chinese Academy of Sciences, Kunming, 650223 Yunnan China

**Keywords:** HIV infections, Virus-host interactions

## Abstract

The reactivation of HIV latency cell will be necessary to curing HIV infection. Although many latency-reversal agents (LRAs) have proven effective to reactivate the latency cell, there is a lack of any systematic analysis of the molecular targets of these LRAs and related pathways in the context of transcriptome. In this study, we performed an integrated analysis of the target profile of bryostatin and transcriptome of the reactivated CD4^+^ T cells after exposing to bryostatin. The result showed a distinct gene expression profile between latency cells and bryostatin reactivated cells. We found bryostatin can target multiple types of protein other than only protein kinase C. Functional network analysis of the target profile and differential expressed genes suggested that bryostatin may activate a few novel pathways such as pyrimidine metabolism, purine metabolism and p53 signaling pathway, besides commonly known pathways DNA replication, cell cycle and so on. The results suggest that bryostatin may reactivate the HIV-latent cells through up-regulation of pyrimidine and purine metabolism or through starting the cell-cycle arrest and apoptosis induced by up-regulation of p53 signaling pathway. Our study provides some novel insights into the role of bryostatin and its affected pathways in controlling HIV latency and reactivation.

## Introduction

Human immunodeficiency virus type 1 (hereafter referred to as “HIV”) is the causative agent of acquired immune deficiency syndrome (AIDS). In the host cells, HIV infection causes to produce viral RNAs and proteins, results in profound functional and morphological changes, and alerts the progression of the host cell cycle. Meanwhile, HIV genomes can integrate to the host cellular DNA as a persistent viral reservoir and harboring replication-competent proviruses with a dormancy state^1^.

Through the development of specific inhibitors of HIV replication, significant progress has been made in the treatment of HIV infection. The success of anti-retroviral (ARV) therapy reduces HIV plasma viremia to an under-detectable level^2^. However, HIV is not eradicated and AIDS patients must remain on ARV drugs for life. A major obstacle to cure AIDS is the HIV latency that is a state of reversibly nonproductive infection in the resting CD4 T cells and possibly other cells^3^. The long-lived reservoir is resistant to ARV therapy and to clearance by the host immune system. Once treatment is stopped, HIV originating from the reservoir lead to re-seed the infection^4^.

The molecular mechanisms of HIV latency are complex and numerous studies have been performed to explain this phenomenon^3,5,6^. In general, a combination of trans-effects and cis-effects governs the activity of HIV promoter transcription making the reservoir the best-established barrier to HIV eradication. Mostly cis-effects reflect the variety of chromatin environments at different sites of integration genome and trans-effects reflect the trans-acting transcription factors within the host cells^5^. For example, heterochromatin of the host genome after HIV infection impairs the gene expression by impeding transcription factors access to the underlying DNA^7^. As the fundamental structural unit of chromatin, nucleosomes are regulated by posttranslational modifications of the histone tails which include phosphorylation, methylation, acetylation, ubiquitylation and poly ADP-ribosylation^8^. Specific combinations of these repressive histone marks have been proposed to govern the chromatin assembly and HIV gene expression^9^. Similarly, the strong repressive state of the HIV latency is correlated with methylation of CpG islands within the HIV promoter and DNA demethylation is associated with re-activation of latent HIV^10^. Another mechanism that can cause HIV transcriptional silence is the transcriptional interference of the HIV promoters and cellular promoters in the integration genome^11^. Depending on the orientation of the HIV provirus relative to the host cells genes, different forms of transcriptional interference occur^12^.

It has become clear that reactivation of HIV latency cells in AIDS patients on ARV therapy will be necessary to curing HIV infection. This is the strategy named “shock and kill” to facilitate a cure for HIV. This strategy administers latency reactivate agents to patients while on ARV therapy in order to induce pro-viral transcription in latently infected cells^13^. Several classes of compounds have been discovered to “Shock” HIV out of latency including; protein kinase C (PKC) agonists^14^, histone deacetylase inhibitors (HDACis)^15^, histone methyltransferase inhibitors (HMTIs)^16^, cytokines, bromodomain inhibitors^17^ and so on.

The PKC agonists is the only one latency-reversal agents (LRAs) class to demonstrate reproducible results across different HIV latency models^18^. Generally, the PKC agonists can be divided into three major chemical families: phorbol esters, including prostratin, 12-deoxyphorbol 13-phenylacetate (DPP) and phorbol 12-myristate 13-acetate (PMA); diterpenes, which include compounds of ingenol; and macrocyclic lactones including bryostatin and its analogs^19^. Among these PKC agonists, bryostatin-1 (hereafter referred to as “bryostatin”) has shown significant potency to revert HIV latency in *ex vivo* experiment using patient cells, compared with other LRAs^20^.

Although these compounds above have proven effective to reactivate the latency cell, the molecular mechanisms underlying the effects are not entirely clear. The activities of these compounds cannot be attributed wholly to a single target and may involve several molecular targets and pathways. Unfortunately, there is no systematic analysis of molecular targets of these LRAs and their related pathway. In this study, we performed an integrated analysis of the target profile of bryostatin and transcriptome of the reactivated CD4^+^ T cells after exposing to bryostatin. The result showed a distinct gene expression profile between the latency cells and bryostatin reactivated cells. Extensive changes of gene expression occurred in the CD4^+^ T cells treated with bryostatin. Furthermore, we found bryostatin can target multiple types of protein other than only PKC. Functional network analysis of the target profile and differential expressed genes (DEGs) suggested that bryostatin may activate a few novel pathways such as pyrimidine metabolism and purine metabolism and p53 signaling pathway, in addition to the commonly known pathways DNA replication, cell cycle, nucleotide excision repair and mismatch repair. These results provide mechanistic insights into the role of bryostatin and its affected pathways in controlling HIV latency and reactivation.

## Results

### Extensive changes of gene expression in CD4^+^ T cell exposed to bryostatin

**Figure 1:**
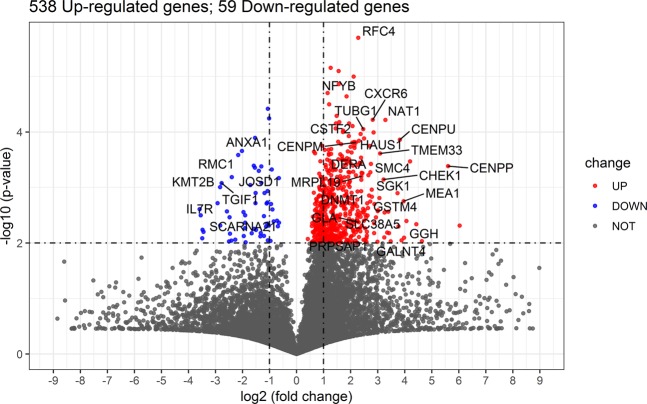
The differential expressed genes in CD4^+^ T cell treated with bryostatin. The x-axis is log_2_ ratio of gene expression levels between the bryostatin stimulated and unstimulated CD4^+^ T cells; the y-axis is P value based on −log_10_. The red and blue dots represent the up-regulated and down-regulated gene (P value < 0.01), respectively; the most significant DEGs (P value < 0.05, AUC >= 0.95 and |log_2_FC| > 2) were labeled with gene symbol.

Compared with the unstimulated CD4^+^ T cell, we identified 597 DEGs (P value< 0.01, Table S1) in the bryostatin stimulated CD4^+^ T cell. In the DEGs, there are 538 up-regulated and 59 down-regulated genes (Fig. 1). It indicates that extensive changes of gene expression in CD4^+^ T cells after exposing to bryostatin. We listed the top 30 most significantly deregulated genes, most of which were up-regulated (Table 1 and labeled in Fig. 1). The expression of genes *CENPP*, *GGH*, *MEA1*, *CENPU*, *SGK*1, *GALNT4*, and *SLC38A5* was upregulated more than 10 folds. In contrast, the expression of genes *KMT2B*, *IL7R* was decreased by over four-fifths. The most obvious DEGs for up-regulation and down-regulation were *CENPP* (FC = 48.57) and *IL7R* (FC = 0.132), respectively.

**Table 1:** The information about the most significantly deregulated genes (|Log_2_ FC| > 2 and AUC > 0.95).

Gene	Fold change	Log_2_ (FC)	AUC	P value	Change trend
*CENPP*	48.5768	5.6022	1.0000	4.12E-04	UP
*GGH*	15.7602	3.9782	0.9753	8.00E-03	UP
*MEA1*	15.5598	3.9597	0.9877	1.76E-03	UP
*CENPU*	14.1561	3.8234	0.9877	1.37E-04	UP
*SGK1*	13.2958	3.7329	0.9753	1.26E-03	UP
*GALNT4*	11.1719	3.4818	0.9753	9.35E-03	UP
*SLC38A5*	10.2226	3.3537	0.9753	6.48E-03	UP
*NAT1*	9.7619	3.2872	0.9877	6.06E-05	UP
*GSTM4*	9.5371	3.2536	0.9506	2.82E-03	UP
*CHEK1*	9.3069	3.2183	0.9506	7.16E-04	UP
*TMEM33*	8.5065	3.0886	0.9877	2.43E-04	UP
*CXCR6*	7.0550	2.8186	0.9753	6.03E-05	UP
*DNMT1*	6.8638	2.7790	0.9630	1.09E-03	UP
*SMC4*	6.7204	2.7485	0.9753	3.76E-04	UP
*TUBG1*	5.5322	2.4678	0.9506	8.85E-05	UP
*MRPL19*	5.2580	2.3945	0.9506	6.28E-04	UP
*CSTF2*	5.1697	2.3701	0.9630	1.08E-04	UP
*HAUS1*	4.9499	2.3074	0.9630	1.44E-04	UP
*PRPSAP1*	4.9473	2.3067	0.9506	7.81E-03	UP
*GLA*	4.9143	2.2970	0.9506	4.22E-03	UP
*RFC4*	4.8528	2.2788	0.9753	2.00E-06	UP
*CENPM*	4.5732	2.1932	0.9630	1.55E-04	UP
*NFYB*	4.3205	2.1112	0.9877	1.01E-05	UP
*DERA*	4.0983	2.0350	0.9753	2.33E-04	UP
*ANXA1*	0.2462	−2.0224	1.0000	2.19E-04	DOWN
*RMC1*	0.2233	−2.1627	0.9753	2.60E-04	DOWN
*JOSD1*	0.1898	−2.3978	0.9506	6.49E-04	DOWN
*SCARNA21*	0.1790	−2.4816	0.9506	3.44E-03	DOWN
*TGIF1*	0.1461	−2.7745	0.9630	8.33E-04	DOWN
*KMT2B*	0.1403	−2.8331	0.9877	9.87E-04	DOWN
*IL7R*	0.1319	−2.9230	0.9877	1.91E-03	DOWN

### The bryostatin treated and unstimulated CD4^+^ T cells showed a distinct gene expression profile

**Figure 2:**
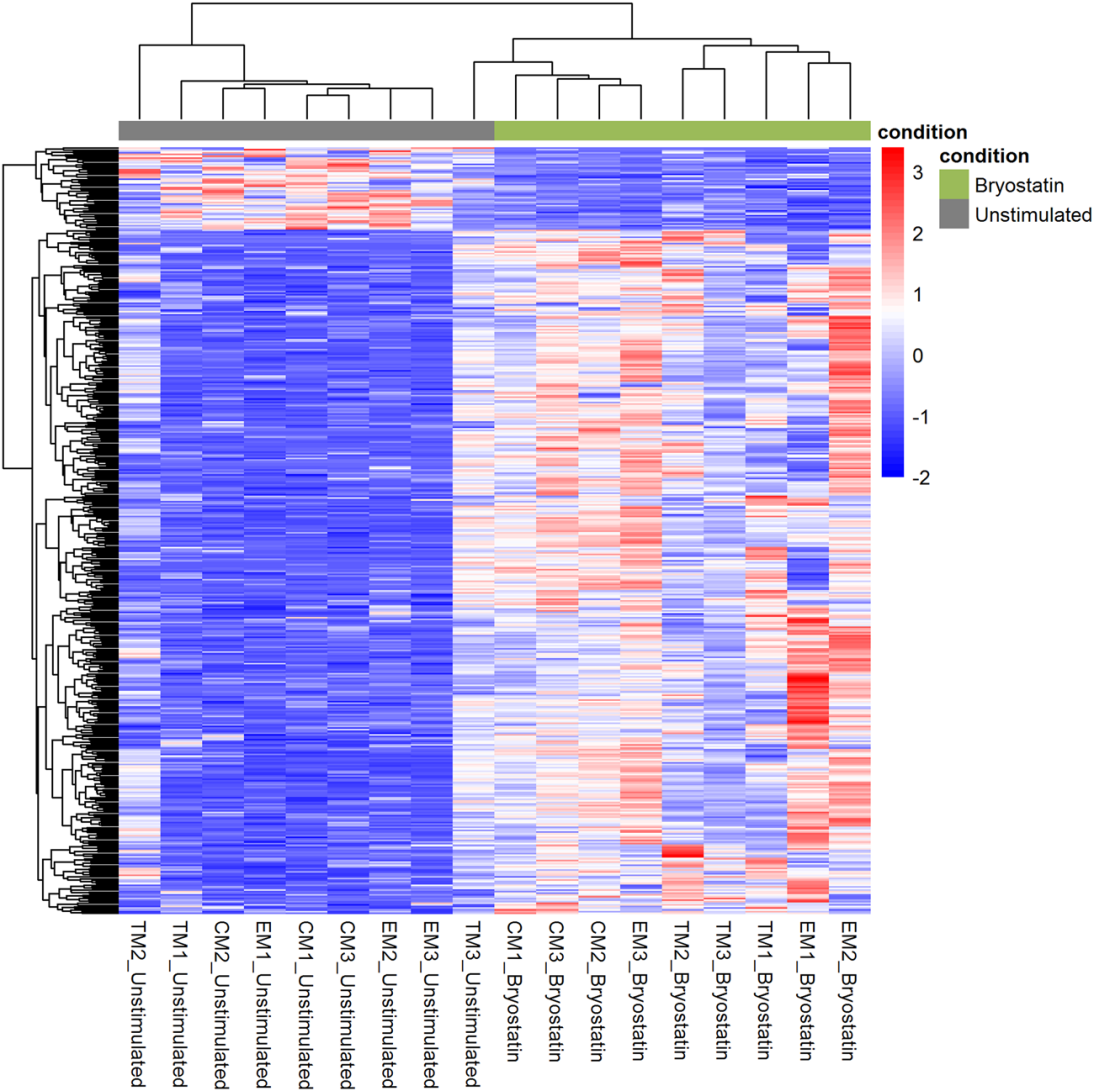
A distinct gene expression profile between the bryostatin stimulated and unstimulated CD4^+^ T cells. The heatmap of 464 significant DEGs (P value < 0.01 and |log_2_FC| > 1) shows the hierarchical clustering of the 18 samples. (**Bryostatin**: bryostatin treated cell; **Unstimulated**: unstimulated CD4^+^ T cells). The color and shade change correspond to the expression value of gene after computing logarithms (log2) and normalization.

To further overview the expression profile of CD4^+^ T cells under two conditions, hierarchical clustering analysis with a heatmap was performed (Fig. 2). The hierarchical clustering was generated using 464 DEGs (P value < 0.01 and |log_2_FC| > 1). In the hierarchical clustering result, 18 samples were divided two distinct categories clearly. The bryostatin treated samples showed a distinct gene expression profile from the that of the unstimulated CD4^+^ T cells, except the sample “TM3_ unstimulated”. Most of these DEGs are up-regulated in the bryostatin treated CD4^+^ T cells.

### Significantly changed pathways in CD4+ cells from latency to reactivation state

**Figure 3:**
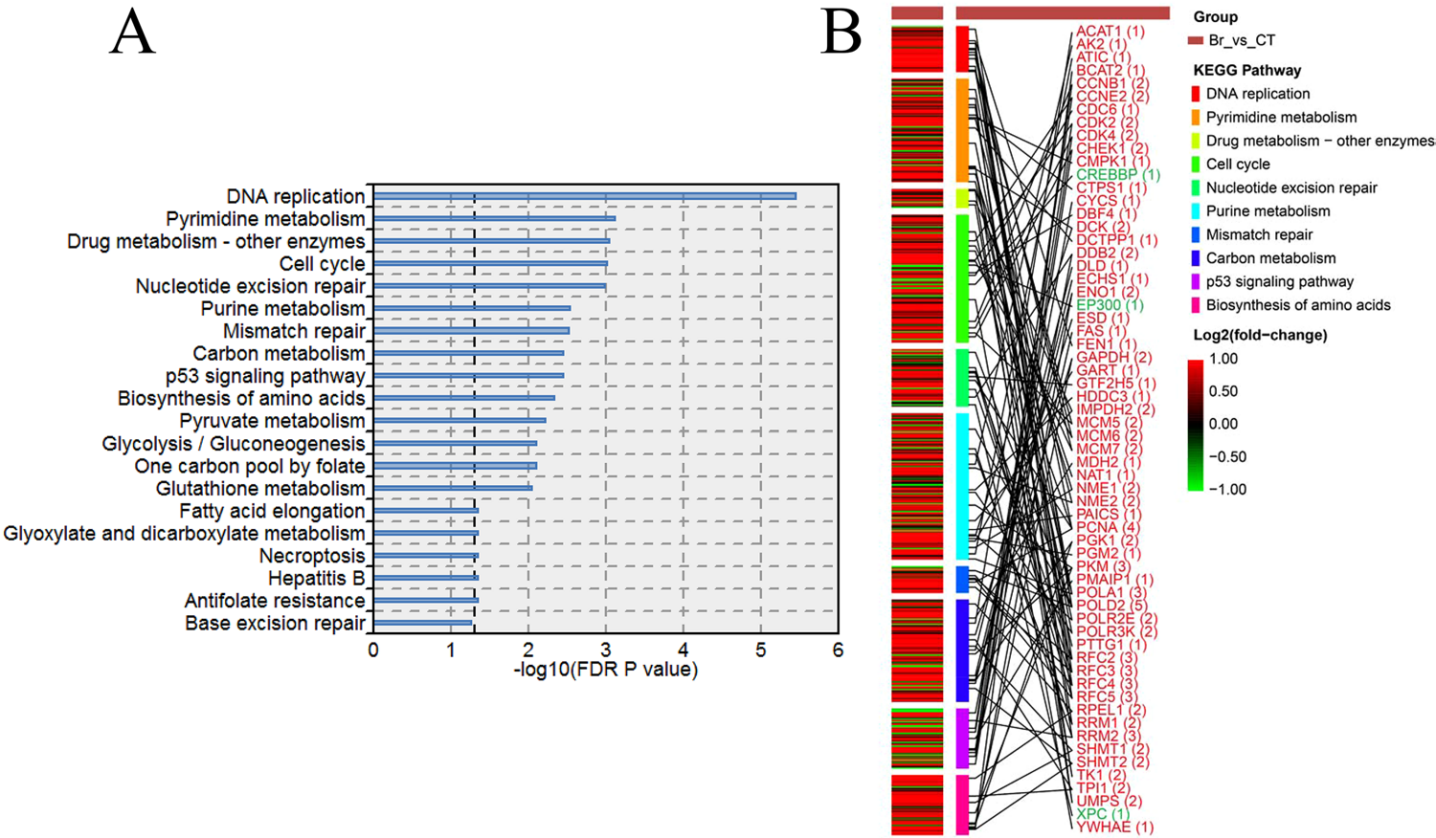
KEGG pathways significantly enriched by DEGs and the network of the top 10 enriched pathways and DEGs. (**A**) The KEGG pathways enriched by DEGs. The line width indicates the enrichment percentage. The dotted line in the box indicates the significance threshold (FDR P value = 0.05). P values were measured by a hypergeometric test. (**B**) The selected top 10 pathways and DEGs for the explanations the treatment effect of bryostatin. The network showed the relationship between the affected pathways and DEGs (|log_2_FC| > 2).

As mentioned above, after treated with bryostatin, CD4^+^ cells were activated and extensive changes of gene expression occurred. A total of 538 and 59 genes were up-regulated and down-regulated, respectively. These significantly DEGs were enriched in 20 pathways revealed by KEGG pathway enrichment analysis and false discovery rate (FDR) adjustment (Fig. 3A). The top changed pathways were DNA replication, pyrimidine metabolism, drug metabolism - other enzymes, cell cycle, nucleotide excision repair, purine metabolism, mismatch repair, carbon metabolism, p53 signaling pathway, and biosynthesis of amino acids. In these pathways, 62 genes were significantly differential expressed (fold change > 2) (Fig. 3B). These genes can perform functions in multiple pathways. For example, *POLD2* gene is involved in five pathways such as purine metabolism, pyrimidine metabolism, DNA replication, nucleotide excision repair and mismatch repair. Another example is *PCNA* gene, which is involved in four pathways as showed in the Fig. 3B. These pathways and involved DEGs provide clear clues for the molecular mechanism of HIV latency reactivation.

### The 17 putative targets of bryostatin predicted by two different methods

**Figure 4:**
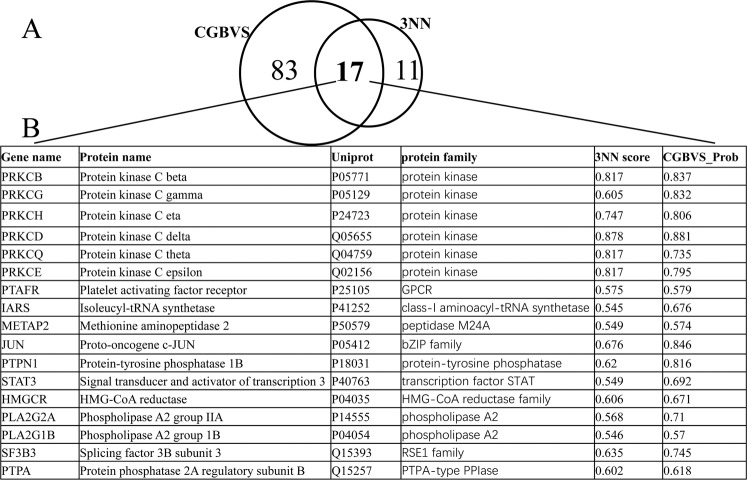
Venn diagram of the predicted targets for bryostatin by the two different methods and the consistent targets. (**A**) Venn diagram of the predicted targets by CGBVS and 3NN. (**B**) Details for the 17 consistent targets predicted by the two methods.

In order to further understand the action mechanism of bryostatin, we predicted the putative targets for bryostatin. Target prediction is still a challenging task so far and plays a critical role in the drug development^21^. It is useful to explore ligand-target interactions and their related bio-chemical mechanisms. We used the two methods CGBVS and 3NN to predict the putative targets of bryostatin. Chemical genomics-based virtual screening (CGBVS) is a proteome-wide method which predicts compound and protein interactions (CPIs) only based on the character description of protein sequence. In contrast, 3NN method is based on ligand similarity. A total of 100 and 28 targets of bryostatin were predicted by CGBVS and 3NN, respectively (Fig. 4A). Among these predicted targets, 17 overlap targets were considered as consistent targets of bryostatin. The 17 overlap targets were *PRKCB*, *PRKCG*, *PRKCH*, *PRKCD*, *PRKCQ*, *PRKCE*, *PTAFR*, *IARS*, *METAP2*, *JUN*, *PTPN1*, *STAT3*, *HMGCR*, *PLA2G2A*, *PLA2G1B*, *SF3B3*, *PTPA* (Fig. 4B). They mainly belong to the protein families of protein kinase, phospholipase A2, GPCR, peptidase M24A and so on. Among these targets, protein kinase C family is the primary known targets of bryostatin. The other protein families are defined as putative targets of bryostatin based on the result of the two methods of CGBVS and 3NN methods. Bryostatin may reactivate the HIV latent CD4^+^ cells through multiple targets and their affected pathways.

### Novel pathways of HIV latency reactivation revealed by functional network between bryostatin targets and DEGs

**Figure 5:**
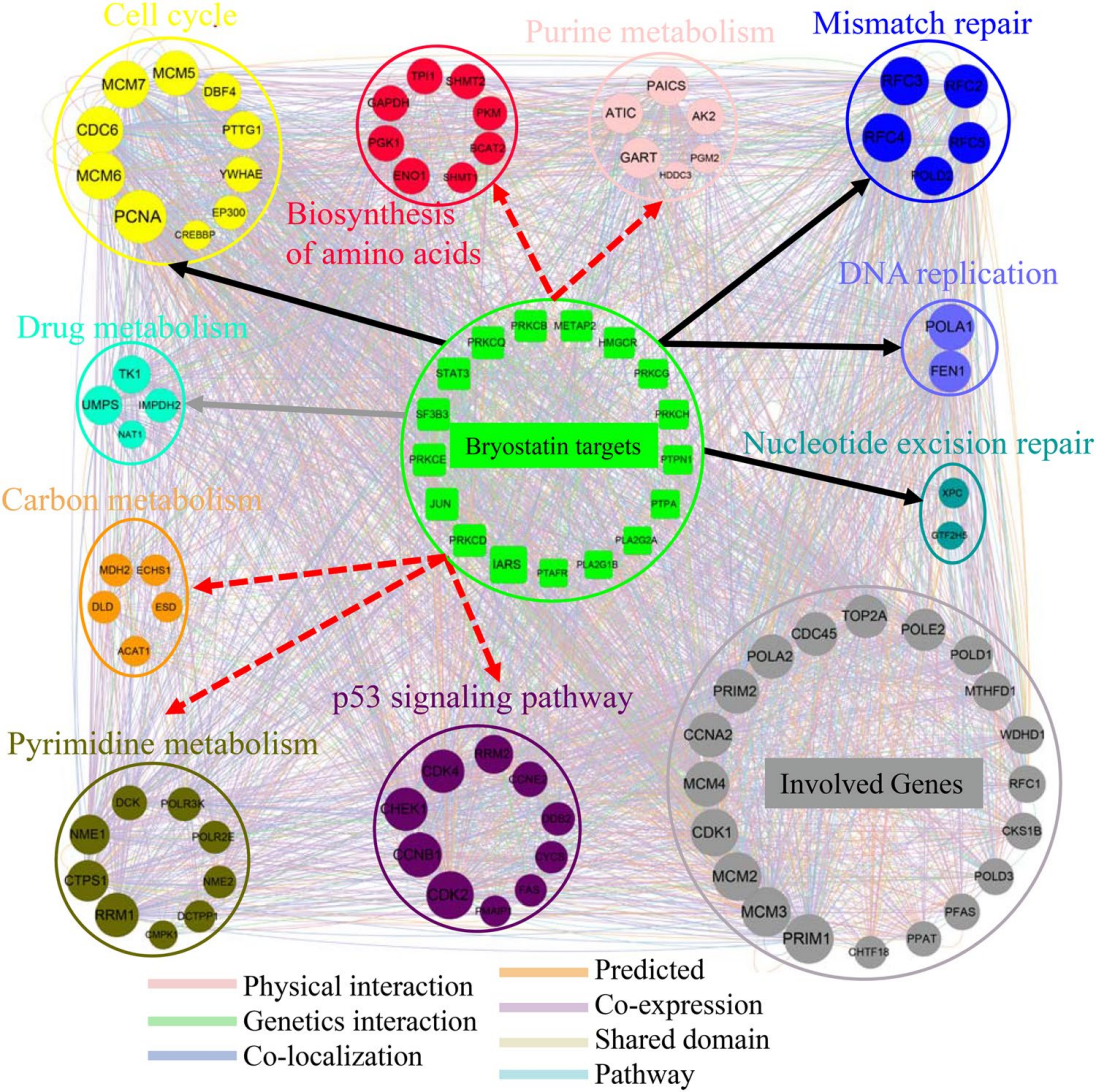
Functional network analysis between the consistent targets of bryostatin and the most DEGs. The network shows the functional interaction between the consistent targets (green rectangle) and most DEGs that are circled and labeled by different pathways. The gray nodes represent the result genes involved in the functional interaction between targets (green rectangle) and DEGs. The black arrows represent the known pathways involved in HIV latent reactivation. The dotted arrows represent the novel pathway revealed by this study. Each colored line represents a different interaction, and the color line width indicates the confidence of the interactions.

KEGG enrichment analysis revealed the top 10 changed pathways and 62 genes in these pathways significantly differential expressed (fold change >2) after exposing to bryostatin. Meanwhile, we predicted 17 consistent targets for bryostatin. To determine whether or how these targets of bryostatin affect the top 10 changed pathways and DEGs, we constructed a functional network between these targets and DEGs using GeneMANIA (Fig. 5). As Fig. 5 showed, the functional network revealed intense interaction between bryostatin targets and DEGs. Based on the KEGG database, the DEGs were labeled by different pathways. We can see clearly that bryostatin reactivate the HIV latent CD4^+^ cells through the effects of multiple targets. These targets affect several well-known pathways involved in the HIV latent reactivation such as DNA replication, cell cycle, nucleotide excision repair, and mismatch repair. Beside those well-known pathways, we also observed some novel pathways are closely linked to HIV latency reactivation (Fig. 5). These novel pathways are p53 signaling pathway and metabolic pathways including pyrimidine metabolism, purine metabolism, carbon metabolism, and biosynthesis of amino acids. They were not reported to be associated with latent HIV activation based on the past studies. The bryostatin targets may affect these metabolic pathways and p53 signaling, and thus play a role in the reactivation of HIV latent CD4^+^cells.

**Figure 6:**
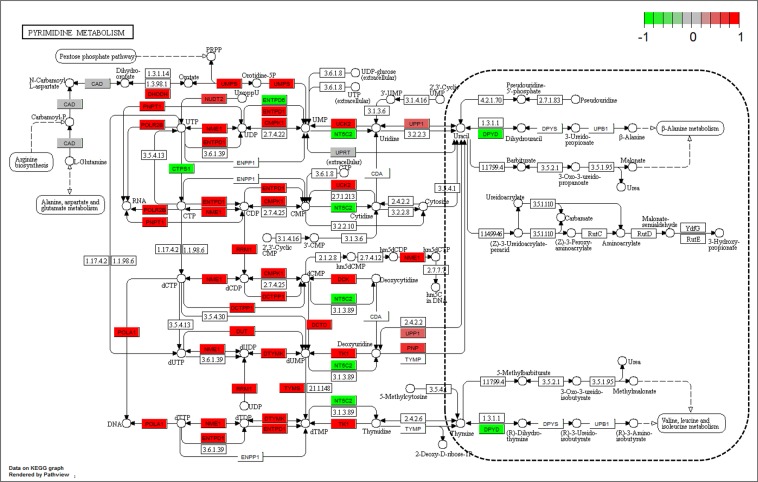
Gene expression profiles of pyrimidine metabolism pathway (hsa00240) in the bryostatin treated CD4^+^ T cells. The red and green colors represent the log_2_(FC) of the corresponding gene in pyrimidine metabolism pathway. The dotted line circles the inactive part of this pathway^50–52^.

**Figure 7:**
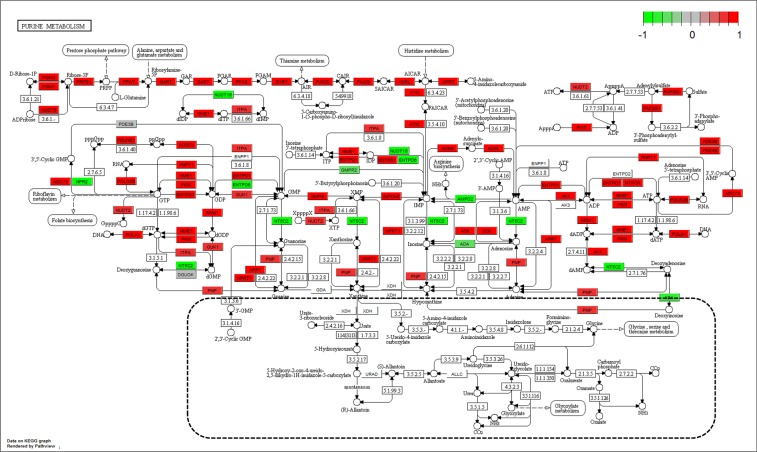
Gene expression profiles of purine metabolism pathway (hsa00230) in the bryostatin treated CD4^+^ T cells. The red and green colors represent the log_2_(FC) of the corresponding gene in purine metabolism pathway. The dotted line circles the inactive part of this pathway^50–52^.

There are more than 30 genes in pyrimidine metabolism pathway highly expressed in the CD4^+^ cells after exposing to bryostatin (Fig. 6). In contrast, only a few genes were down-regulated. From the upstream genes (*UMPS, DHODH, DCK, NME1, RRM1, PNP, etc*.) to downstream genes (*POLA1, POLR2E, etc*.) of this pathway, mostly of genes were highly active. Interestingly, we have found that the half parts of DNA synthesis and RNA transcription are highly active. In contrast, the other parts related to the catabolism and amino acid synthesis are under extreme inhibition and haven’t changed much in gene expression. Similarly, more than 50 genes in purine metabolism pathway were highly expressed in the CD4^+^ cells after exposing to bryostatin (Fig. 7). The pattern of gene expression was also similar to that of the pyrimidine metabolism pathway. This differential gene expression may drive the metabolic flux toward to the parts of DNA synthesis and RNA transcription, other than the parts related to the catabolism and amino acid synthesis.

**Figure 8:**
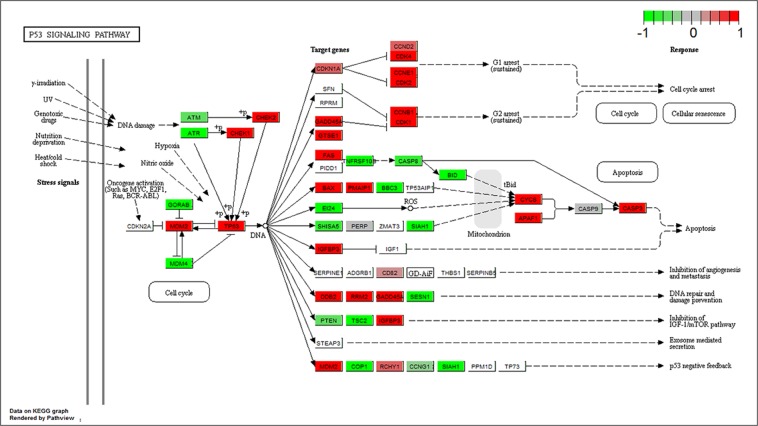
Gene expression profiles of p53 signaling pathway (hsa04115) in the bryostatin treated CD4^+^ T cells. The red and green colors represent the log_2_(FC) of the corresponding gene in p53 signaling pathway^50–52^.

The p53 signaling pathway is a key factor that helps to conserve the stability of the genome. It regulates a wide variety of cellular processes including apoptosis, senescence, cell cycle arrest, differentiation and so on. The p53 signaling pathway is significantly changed after exposing to bryostatin (Fig. 8). Genes *CDK1*, *CDK2*, *CDK4*, *CCNE1*, *CCNB1* were up-regulated, which are contributes to the cell cycle arrest. Gene *CASP3* is up-regulated, which will lead to apoptosis. Therefore, the cell cycle arrest and apoptosis may have a potential role in the reactivation of HIV latency.

## Discussion

The latent reservoir is mostly found in resting memory CD4^+^ T cells^22,23^. The most worrisome reservoir consists of latently infected resting memory CD4^+^ T cells carrying integrated HIV-1 DNA. Bryostatin has shown significant potency to revert HIV latency in *ex vivo* experiment using patient cells, compared with other LRAs^20^. In this study, we focused to characterize the transcriptome in both the bryostatin treated and unstimulated HIV latent CD4^+^ T cells. Bryostatin stimulation will lead to reactivation of HIV-latent CD4^+^ T cells. To further confirm the change of HIV gene expression before and after reactivation, we checked the study that generated and provided the data^24^. The result showed that bryostatin significantly reactivated the latency cell compared with unstimulated condition (Fig. S1). And bryostatin has the highest efficiency at latency reversal among LRAs. Furthermore, as the importance of interleukins in the reactivation, we checked the gene expression for all interleukins and their receptors. We showed the genes that expressed in at least one sample (Fig. S2). The result indicates that the expression of interleukins and their receptors were heterogeneous. Only three genes IL7R, IL2RA and IL2RA were differential expressed between unstimulated and bryostatin treated samples. IL7R was down-regulated in the bryostatin treated samples. However, IL2RA and IL2RA were up-regulated in the bryostatin treated samples. Most interleukins and their receptors did not show differential expression, which suggests interleukins and their receptors (including IL7R) may not affect ART efficiency in this study.

Accompanying the reactivation of HIV latency, we found extensive changes of gene expression. We identified a total of 597 DEGs, and most of DEGs are up-regulated. Among the top 30 DEGs, the genes *CXCR6*, *DNMT1* and *JOSD1* had studies on the HIV infection^25–27^, but had no reports on the HIV latency-reversal. Moreover, genes *CENPP*, *CENPU* and *CENPM*, as the component of the centromere complex (nucleosome-associated), were all from centromere protein (CENP) family and upregulated in the HIV latent CD4^+^ T cells treated by bryostatin. Specifically, the gene *CENPP* was the component of CENPA (Histone H3-Like Centromeric Protein A)-CAD (nucleosome distal) complex, and the genes *CENPU/M* were the component of CENPA-NAC (nucleosome-associated) complex^28^. Cellular chromatin provides essential structure to the viral genome and is necessary for successful completion of the HIV life cycle. HIV can disrupt host cellular chromatin during heterochromatin integration and latent reactivation in unique centromere protein (CENPP, CENPU and CENPM) ways. These upregulated genes in the CD4^+^ T cells stimulated by bryostatin may play an important role in the HIV latency-reversal.

The biochemical mechanisms involved in the induction of pro-viral transcription in latent HIV-infected CD4^+^ T cells have not been fully defined. With the development of ARV therapy, it is import to understand how HIV infection changes the host cells pathways and metabolism. In our study, we found that pyrimidine metabolism, purine metabolism, and p53 signaling pathways are very closely related to HIV latency. Purines are key components of cellular energy systems (eg, ATP and NAD), signaling (eg, GTP, cAMP, and cGMP), and, along with pyrimidines, RNA and DNA production^29,30^. Therefore, pyrimidines and purines are crucial for nucleic acid biosynthesis, but also for lipid and carbohydrate. Previous studies revealed that pyrimidine and purine analogues were used as antiviral and immunosuppressive agents^31,32^. For example, arabinosylcytosine (ara-C), 5-fluorouracil (5-FU), and azidothymidine(AZT) have been used to target HIV reverse transcriptase^32^. In general, HIV infection will seriously impair the capability of T cells to synthesize the purine and pyrimidine ribonucleotide intermediates^33^. The shortage of these intermediates obstructs the HIV-infected cells to complete their cycle, thus turns the cell into latent state^33^. Therefore, bryostatin stimulates the latent cell and then results in up-regulation of pyrimidine and purine metabolism, which facilitates reactivation of the latent HIV infected cell.

P53 signaling pathway, a sensor for a broad range of cellular stresses, involved in several different aspects of cell cycle arrest, apoptosis, control of genome integrity, and DNA repair^34^. Cell cycle arrest and apoptosis are the most prominent outcomes of p53 activation^35^. In this study, p53 and its downstream genes (*CCNB1*, *CDK1*, *CCNE1*, *CKK2*, *CDK4*, and *CASP3*) were highly up-regulated (Fig. 8). The up-regulated genes *CCNB1* and *CDK1* will result in the sustained G2 arrest. Similarly, the up-regulated genes *CCNE1*, *CKK2*, and *CDK4* will lead to the sustained G1 arrest. The up-regulated gene *CASP3* will induce apoptosis. Therefore, we assume that bryostatin may lead to cell cycle arrest and apoptosis in the process of reactivation of latent HIV infected CD4^+^ cell. Previous studies indicate that latent HIV-1 can sense the apoptosis of its host cell and responds by completing its replication cycle^36,37^. Another study revealed that G2 arrest of cell cycle induced HIV-1 transcriptional activation^38^. Together with previous studies, our results suggest that the reactivation of HIV latent cell, responding to bryostatin, may be partly due to the cell-cycle arrest and apoptosis induced by up-regulation of p53 signaling pathway.

It is worth noting that bryostatin, a potent activator of protein kinase C, may be involved in mitochondrial dysfunction^39^. Protein kinase C activation can induce mitochondrial dysfunction^40–42^. Protein p53 also can influence mitochondrial function^43,44^. Pyrimidine biosynthesis links mitochondrial respiration to the p53 pathway^45^. Therefore, mitochondrial dysfunction may be involved in the process that bryostatin reactivates the HIV-latent cell through up-regulation of pyrimidine and purine metabolism, and p53 signaling pathway.

In summary, we firstly revealed the extensive changes of gene expression in the process of HIV latent cells, responding to bryostatin. Hundreds of DEGs were identified and enriched both in the well-known and novel pathways. Secondly, we predicted 17 putative targets for bryostatin by two different methods. The 17 targets belong to multiple protein families including protein kinase, phospholipase A2, GPCR, peptidase M24A and so on. Finally, we discovered three novel pathways of pyrimidine metabolism, purine metabolism and p53 signaling important for the reactivation of HIV latency through functional network analysis of the bryostatin targets and DEGs. We assume that bryostatin reactivates the HIV-latent cell through up-regulation of pyrimidine and purine metabolism or through starting the cell-cycle arrest and apoptosis induced by up-regulation of p53 signaling pathway. Our study provides some novel insights into the role of bryostatin and its affected pathway in controlling HIV latency and reactivation.

## Methods

### Expression data collection and preprocessing

The CD4^+^ T cells expression data (GSE94150) was downloaded from Gene Expression Omnibus (GEO). The dataset was generated from the Illumina HiSeq. 2500 (Homo sapiens) and included 36 samples. The information for all samples was shown in Table S2. We described the key information of the samples here. All details can be found from https://www.ncbi.nlm.nih.gov/geo/query/acc.cgi?acc=GSE94150 and reference^24^. The CD4^+^ T cells were isolated from a cohort of Florida HIV-infected subjects that have been on successful ART for >36 months with a viral load ≤50 copies/mL. The CD4^+^ T cells were divided into three subsets namely central memory (T_CM_), transitional memory (T_TM_) and effector memory (T_EM_) CD4^+^ T cells. The memory CD4^+^ T cell T_CM_, T_TM_, and T_EM_ subsets were stimulated for 24 h using different classes of agents including PKC activators (50 nM Bryostatin), gamma-c cytokines (10 ng/ml IL-15) and PMA+Ionomycin (100 ng/ml PMA and 1 μg/ml ionomycin). RNA-Seq was performed on the unstimulated and 24h-stimulated subsets. Unstimulated cells from the same donors were included as pre-reactivation baseline controls. Of 36 samples, 27 samples were stimulated with Bryostatin-1, IL-15 and PMA+Ionomycin, respectively. The other 9 samples were unstimulated. Our aim is to study transcriptional responses of CD4^+^ T cells to the bryostatin-1 and 18 samples were retained (9 bryostatin-1 stimulated and 9 unstimulated CD4^+^ T cells). The raw high-throughput mRNA sequencing reads was downloaded and analyzed by HISAT^46^, StringTie^47^ and Ballgown^48^ protocol. Firstly, the raw RNA-seq reads were mapped to the reference human genome using HISTA. Then, the alignments were passed to StingTie, which assembles and merges together all the gene structures found in any of the samples. In order to re-estimate the transcript abundance using the merged structures, the merged transcripts were fed back to StringTie again. Finally, Ballgown groups the all transcripts and abundances from StringTie by stimulated condition and determines which genes and transcripts are differentially expressed between conditions. The R v3.5.0 and Bioconductor library were used to analyze the expression data.

### KEGG pathways and biological processes analysis

We used the functions enrichKEGG and enrichGOR in clusterProfiler R package^49^ to perform KEGG pathways^50–52^ and biological processes enrichment analysis. A pathway or process with a P value ≤ 0.05 was considered to be significantly enriched. The enriched pathways and processes were visualized using custom written R code. We selected top 10 significantly pathways affected by the compound bryostatin for further study. Therefore, we used the pathview R package^53^ to display the expression profiles of the corresponding genes in these pathways. This package could provide the links between genes and pathways based on the KEGG pathways.

### Prediction of the targets of latency-reversal agent bryostatin

In order to understand the action mechanism of LRA bryostatin, two methods of CGBVS^54,55^ and 3NN^56^ were used to predict the targets of bryostatin. The CGBVS is a chemical genomics-based virtual screening method for target prediction^54^. CGBVS predicts compound-protein interactions (CPIs) by using machine learn only based protein sequence rather than three-dimensional structures. The 3NN is a ligand-based similarity rankings method for target prediction^56^. 3NN method uses the 3 most similar scores (or their average value) between the query and the ligands sets in reference library to rank possible targets. We used venn diagram to show the number of target genes predicted by the two methods. Overlap targets were defined as the consistent targets of bryostatin.

### Functional interaction network analysis

GeneMANIA^57^ plug-in of Cytoscape^58^ was applied to identify the functional interaction between the consistent targets of bryostatin and most DEGs. We used the consistent targets of bryostatin and most DEGs as input parameters. GeneMANIA (http://www.genemania.org)^59^ can find other genes that are related to the set of input genes and produce a functional association network based on their relationships, such as pathways, co-expression, co-localization, genetic interaction, physical interaction, shared protein domains and so on, based on the published literature. Next, we used Cytoscape v3.2.1 to visualize the network.

## Supplementary information

Supplementary Information S1.

Supplementary Dataset 1.

## Data Availability

All data analyzed during this study were described the methods and downloaded from https://www.ncbi.nlm.nih.gov/geo/query/acc.cgi?acc=GSE94150.
