# Supplementary material for: Novel pathways of HIV latency reactivation revealed by integrated analysis of transcriptome and target profile of bryostatin
Source: Sci Rep. 2020 Feb 26;10:3511. doi: 10.1038/s41598-020-60614-1 (PMC7044323; doi:10.1038/s41598-020-60614-1)
Supplement: Supplementary file 1 — Supplementary Information S1. [file 41598_2020_60614_MOESM1_ESM.docx]

**Supplementary Information for**

**Novel pathways of** **HIV** **latency reactivation revealed by integrated analysis of** **transcriptome and target profile of bryostatin**

Bing-xiang Li^1,#^, Han Zhang^1,#^, Yubin Liu^3,#^, Ya Li^4,#^, Jun-juan Zheng^5^, Wen-Xing Li^5^, Kai Feng^1^, Ming Sun^1^*, Shao-Xing Dai^2^*

^1^Institute of Medical Biology, Peking Union Medical College and Chinese Academy of Medical Sciences, Kunming, China

^2^Yunnan Key Laboratory of Primate Biomedicine Research, Institute of Primate Translational Medicine, Kunming University of Science and Technology, Kunming 650500, Yunnan, China

^3^Department of Infectious Diseases, Peking Union Medical College Hospital, Peking Union Medical College and Chinese Academy of Medical Sciences, Beijing, China

^4^Yunnan Key Laboratory of Laboratory Medicine, Yunnan Institute of Laboratory Diagnosis, Department of Clinical Laboratory, The First Affiliated Hospital of Kunming Medical University, Kunming650032, Yunnan, China

^5^State Key Laboratory of Genetic Resources and Evolution, Kunming Institute of Zoology, Chinese Academy of Sciences, Kunming 650223, Yunnan, China

**#These authors contributed equally to this work**

***Correspondence:**

**Ming Sun**, PhD

Institute of Medical Biology, Peking Union Medical College and Chinese Academy of Medical Sciences, Kunming 650118, China; E-mail: sunming@imbcams.com.cn

**Shao-Xing Dai,** PhD

Institute of Primate Translational Medicine, Kunming University of Science and Technology, Kunming 650500, China; E-mail: daishx08@gmail.com


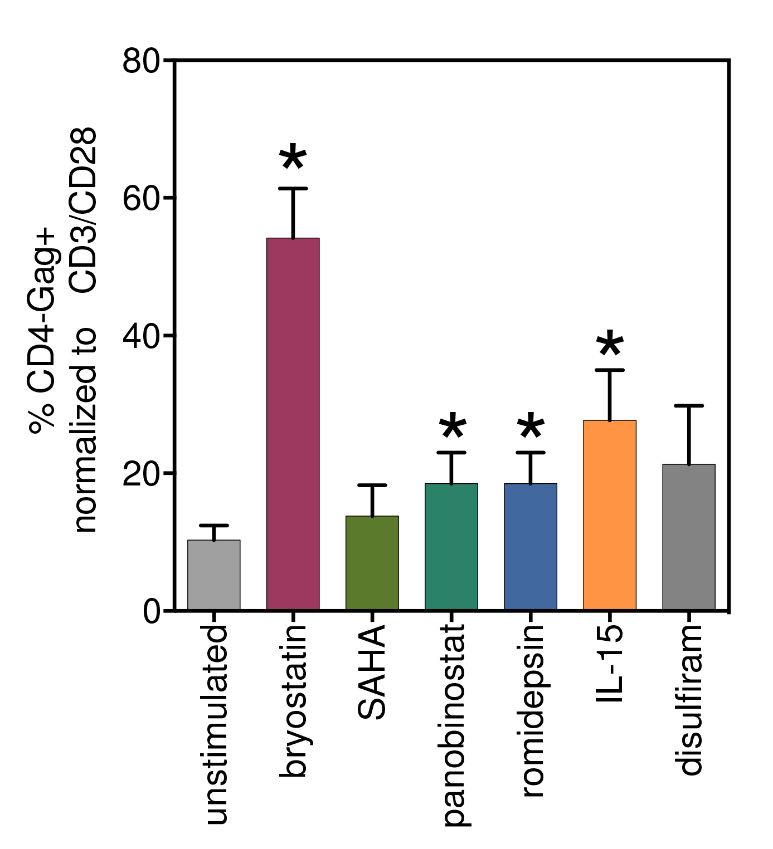


**Figure S1. This figure is from the Figure 7 in the study [1]**. This figure showed the percentages of CD4-Gag^+^ cells within total memory CD4^+^ T cells after exposure to different compounds (LRAs) were normalized to the positive control 1 μg/mL αCD3/CD28. LRA concentrations shown in the original figure. Stars indicate p-value < 0.05 (Wilcoxon rank sum test-paired) for the drug compared to unstimulated controls. LRAs trigger different quantitative responses in memory CD4+ T cell subsets.

**
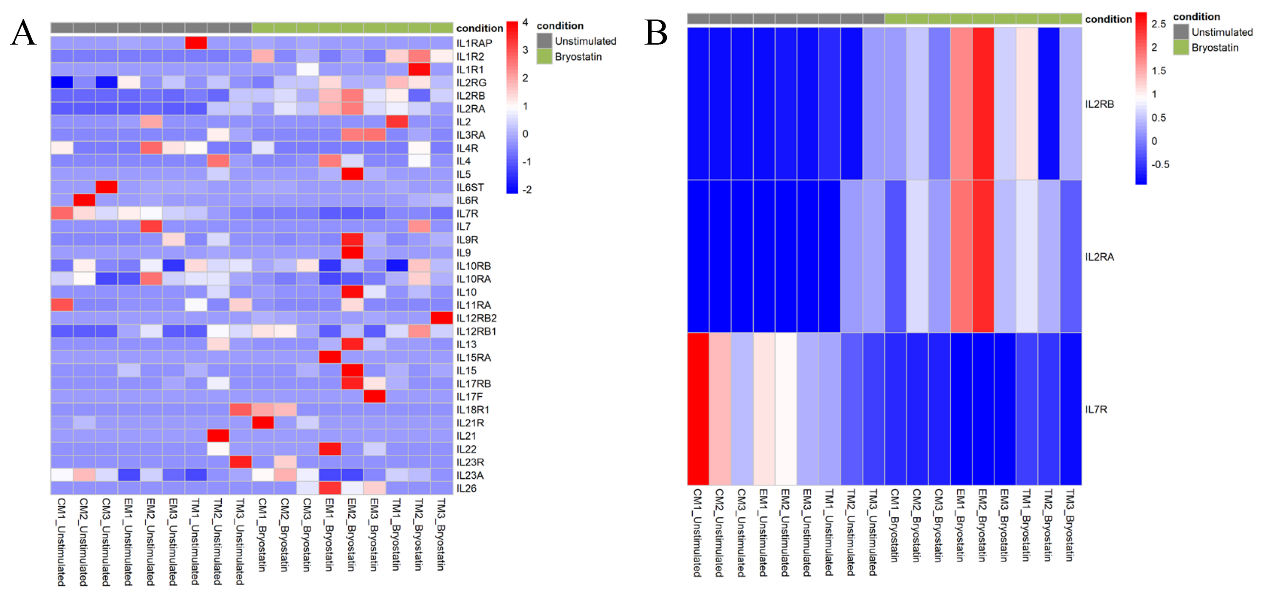
**

**Figure S2.** The heatmap of gene expression for interleukins and their receptors. We showed the genes that expressed in at least one sample. The color and shade change correspond to the expression value of gene after computing logarithms (log2) and normalization.

**Reference**

1. Kulpa, D.A., et al., *Differentiation to an effector memory phenotype potentiates HIV-l latency reversal in CD4+ T cells.* J Virol, 2019.
